# Mumps outbreak in university students: first detection of mumps virus genotype F in Borneo

**DOI:** 10.1186/s41182-022-00411-w

**Published:** 2022-03-02

**Authors:** Siat Yee Fong, Daisuke Mori, Jecelyn Leaslie John, Nelbon Giloi, Mohammad Saffree Jeffree, Kamruddin Ahmed

**Affiliations:** 1 Borneo Medical and Health Research Centre, Faculty of Medicine and Health Sciences, Universiti Malaysia Sabah, Jalan UMS, 88400 Kota Kinabalu, Sabah Malaysia; 2 Department of Biomedical Sciences, Faculty of Medicine and Health Sciences, Universiti Malaysia Sabah, Kota Kinabalu, Sabah Malaysia; 3 Department of Pathology and Microbiology, Faculty of Medicine and Health Sciences, Universiti Malaysia Sabah, Kota Kinabalu, Sabah Malaysia; 4 Department of Health Sciences, School of Medicine, Kyushu University, Fukuoka, Japan; 5 Department of Community and Family Medicine, Faculty of Medicine and Health Sciences, Universiti Malaysia Sabah, Kota Kinabalu, Sabah Malaysia

**Keywords:** Mumps, Outbreak, Genotype, Sabah

## Abstract

**Background:**

In October 2016, a mumps outbreak occurred among the students living in the on-campus dormitory of a public university located in Kota Kinabalu, Sabah, Malaysia. This study aimed to investigate the outbreak and identify the genotype of the mumps virus (MuV) strain that was involved in the outbreak.

**Main body:**

During the outbreak, one 21-year-old and four 20-year-old males staying in the same dormitory building were reported to have developed symptoms of mumps. Of these, two students were available during the investigation for sample collection to detect MuV by reverse transcription polymerase chain reaction (RT-PCR) of the 639-bp fragment encompassing the entire small hydrophobic (SH) gene. Nucleotide sequencing of the amplicon and phylogenetic analysis using the neighbor-joining method was performed to determine the MuV genotype. Of the two buccal swab samples, one was positive for MuV. The MuV strain in this sample belonged to genotype F and it was clustered together with genotype F strains from China with 96.84–99.68% nucleotide identity.

**Conclusions:**

Genotype F has limited circulation and is endemic in mainland China. Genotype F strains occasionally reported from other countries were epidemiologically linked to China. This study is the first to report a case of genotype F MuV in Malaysia and no epidemiological link could be established with mainland China. The results provide important information that can assist in strategic planning to improve the prevention and control of mumps infection in Malaysia.

## Background

Mumps is an acute viral illness that mainly affects young children, adolescents and young adults, characterized by fever and swelling of the parotid glands [1]. Mumps may lead to serious complications such as orchitis, meningitis, and encephalitis [2]. Mumps virus (MuV) is a member of the genus *Rubulavirus* belonging to the family *Paramyxoviridae* that only infects humans [3]. MuV has a singled-stranded, negative-sense RNA genome consisting of 15,384 nucleotides [3]. The genome encodes two surface glycoproteins (fusion [F] and haemagglutinin-neuraminidase [HN]), four core proteins (nucleoprotein [NP], virion/phosphoprotein [V/P], matrix [M] and large [L] protein), and the membrane-associated small hydrophobic (SH) protein [4]. The SH gene is the most variable segment of the MuV genome and is recommended for genotyping [4]. Based on the sequence analysis of all 316 nucleotides of the SH gene, there are 12 mumps genotypes A to N (excluding E and M) that are currently recognized [5]. Up-to-date information on the distribution and circulation of MuV genotypes is important for the development and improvement of mumps epidemiology and national vaccination policy. However, the MuV genotypes circulating in Malaysia have not been studied thus far. Here, we report on an outbreak of mumps among students living in an on-campus dormitory at a public university located in Kota Kinabalu, Sabah, Malaysia. The genotype of the detected MuV strain was also investigated in this study.

## Materials and methods

On October 27, 2016, the Kota Kinabalu Area Health Office was notified of a mumps outbreak involving students at a public university in Kota Kinabalu, Sabah, who were living in an on-campus dormitory. A team was formed from the Faculty of Medicine and Health Sciences, Universiti Malaysia Sabah and dispatched to investigate the outbreak in the concerned dormitory.

### Case findings

**Fig. 1:**
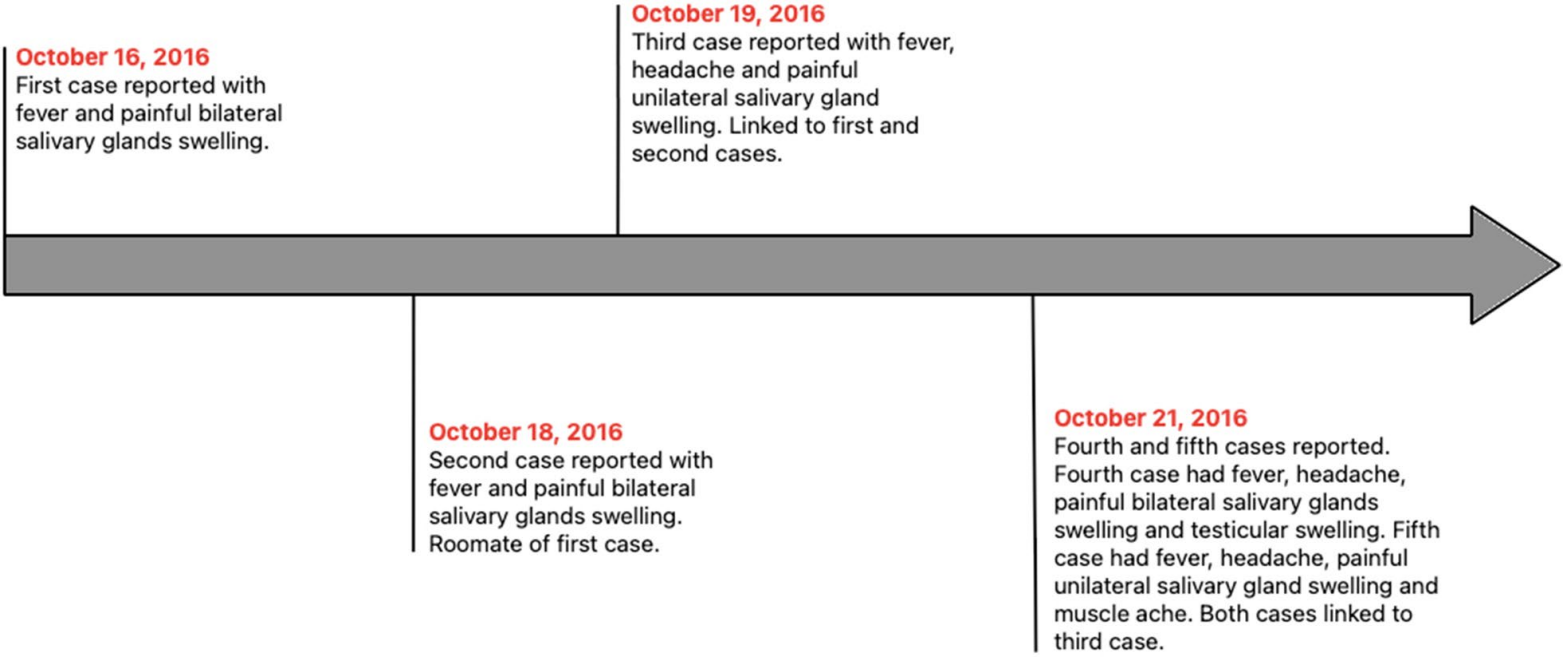
Timeline of events during the outbreak

All students in this study were interviewed to obtain information on their mumps vaccination status and travel history. The primary case was that of a 21-year-old male third-year medical student who developed fever and painful bilateral salivary gland swelling on October 16, 2016. He had no history of interaction with mumps patients or travel outside Sabah. On October 18, 2016, one of his three roommates developed the same symptoms, constituting the second case. On October 19, 2016, the third student developed fever, headache, and painful unilateral salivary gland swelling. This student resided in another room but often visited the first and second cases in their shared room. The third student’s roommates were not affected. The fourth and fifth students developed symptoms on October 21, 2016. The fourth student had fever, headache, and painful bilateral salivary gland swelling, and was the only one with testicular pain and swelling. He stayed in another room but had driven the third student to seek treatment on October 19, 2016. The fifth student, who also resided in a separate room, had fever, headache, painful unilateral salivary gland swelling, and muscle ache. He was a close friend of the third student and often spent time in the third student’s room. The roommates of the fourth and fifth students were not tested and displayed no symptoms. The fifth student’s dormitory room was located opposite to the first and second students’ rooms. Other than the first student, all students were 20-year-old male second-year medical students at the same university faculty. All five cases resolved spontaneously without complications and no cases were reported thereafter. The timeline of the outbreak is summarized in Fig. 1.

### RT-PCR for mumps virus detection

Two buccal swab samples were collected from the fourth and fifth students. The other students were either not present in the dormitory at the time of investigation or had recovered from mumps. Genomic RNA was extracted using a QIAmp Viral RNA Mini kit (Qiagen, Hilden, Germany) according to manufacturer’s instructions. Extracted RNA was reverse transcribed to cDNA for the initial detection of *Paramyxoviridae* viruses by reverse transcription polymerase chain reaction (RT-PCR), targeting the L gene [6]. Then, nested PCR amplification of the 639-bp fragment encompassing the entire SH gene was performed using two sets of primers to specifically detect MuV. Primers SH1 (5′AGTAGTGTCGATGATCTCAT) and SH2R (5′GCTCAAGCCTTGATCATTGA) at 10 pmol each were used for the first round of PCR, while primers SH3 (5′GTCGATGATCTCATCAGGTAC) and SH4R (5′AGCTCACCTAAAGTGACAAT) at 25 pmol each were used for the nested PCR. The PCR amplification conditions were: initial denaturation at 95 °C for 2 min, followed by 25 cycles of denaturation at 95 °C for 1 min, annealing at 50 °C for 1.5 min (first round) or 55 °C for 1.5 min (nested PCR), extension at 72 °C for 2 min and a final extension step at 72 °C for 5 min [7]. All protocols were carried out in accordance with the guidelines and regulations of the National Committee for Clinical Research, Ministry of Health, Malaysia [8].

### Nucleotide sequencing and phylogenetic analysis

The purified amplicon of the RT-PCR-positive sample was sequenced using an ABI Prism 3130 Genetic Analyzer (Applied Biosystems, Foster City, CA, USA) according to manufacturer’s instructions. Basic Local Alignment Search Tool (BLAST; www.ncbi.nlm.nih.gov/blast) was used to identify the virus and genotype. Nucleotide sequences of the SH gene of other MuV strains were retrieved from GenBank. Multiple sequence alignment was conducted using ClustalW2 (www.ebi.ac.uk/clustalw). The phylogenetic analysis was performed based on the neighbor-joining method using MEGA 7.0 software (https://www.megasoftware.net). A bootstrap analysis of 1,000 replicates was performed to test the reliability of the branching pattern. The nucleotide sequence analyzed in this study has been submitted to the DNA Data Bank of Japan (DDBJ).

## Results

This study investigated and characterized the genotype of an MuV strain detected during a mumps outbreak. The students in this study were all born before 2002 when measles-mumps-rubella (MMR) vaccine was first introduced in the National Immunisation Programme of Malaysia. From interviews, none of the students were vaccinated against MuV and they had no history of travelling outside of Malaysia or exposed to any known mumps patients. Although we used a very sensitive method which could detect 1–10 copies of MuV cDNA, only one sample (fifth student) tested positive. The student’s infection was resolving at the time of sample collection, and this may have resulted in the absence of MuV in the sample of the fourth student.

**Fig. 2:**
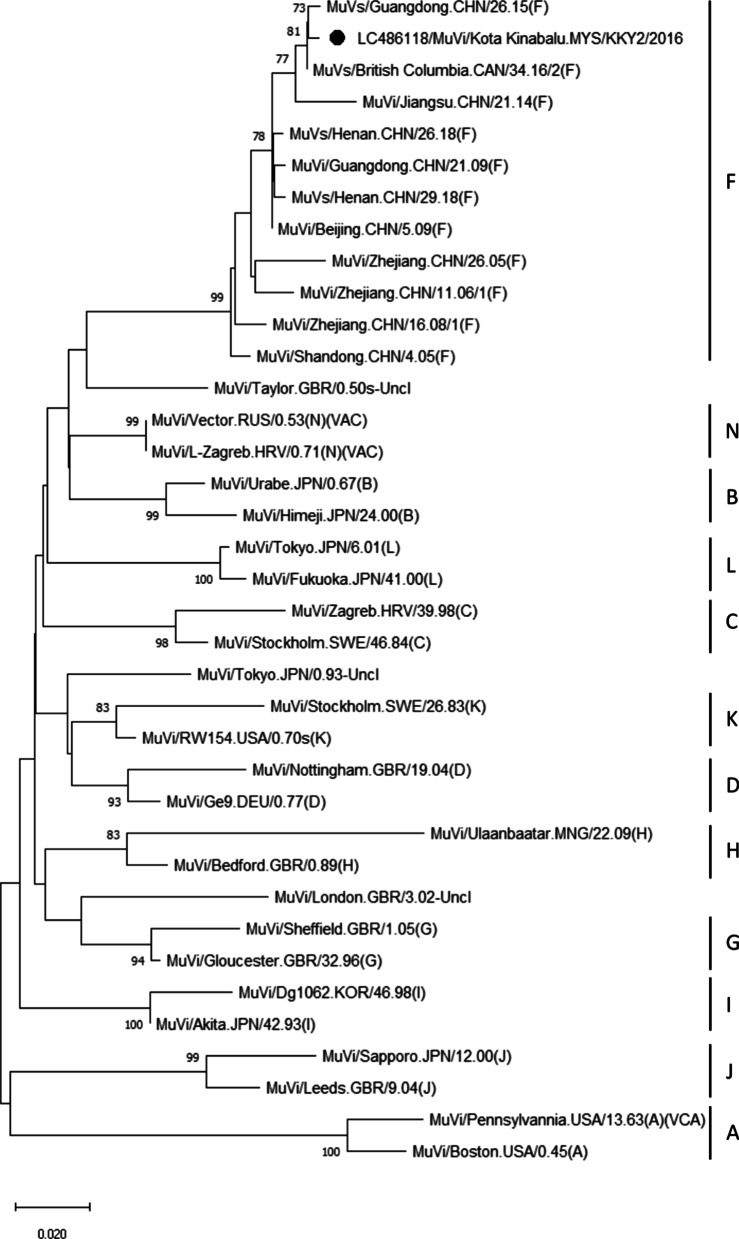
Phylogenetic tree constructed using the 639-bp sequence encompassing the entire SH gene by neighbor-joining method. The strain analyzed in this study is marked with a filled circle. Our strain belonged to genotype F and clustered together with genotype F strains from China and Canada. The number adjacent to the node represents the bootstrap value; values < 70% are not shown. The scale bar at the bottom indicates the genetic distance expressed as nucleotide substitutions per site. The nucleotide sequence of our strain has been submitted to the DNA Data Bank of Japan (DDBJ) with accession no. LC486118

Genotyping of the SH gene revealed that the strain detected in this study belonged to genotype F and clustered together with ten genotype F MuV strains from China and one from Canada (Fig. 2) with a significant bootstrap value. The nucleotide identity among these strains were 96.84–99.68%.

## Discussion

There is a differential distribution of MuV genotypes worldwide but multiple genotypes can co-circulate simultaneously within a geographical region, leading to temporal shifts in their distribution [4]. Genotypes C, D, E, G and H are common in Europe and the United States of America, whereas genotypes B, F and I are more prevalent in Asian countries [4]. For instance, a recent mumps outbreak in the United States of America was caused by genotype G [9], whereas in China, genotype F MuV was responsible for an outbreak in Henan province in 2016 [10]. Genotype F has limited circulation and has been endemic in China since its discovery in 1995 [10, 11]. Although genotype F has occasionally been found in other countries in North America, Europe and Asia, the MuV strains from these countries were epidemiologically linked to China [12, 13]. Interestingly, the MuV strain detected in this study did not only cluster with strains from China but also phylogenetically related to a strain from Canada, where the patient had a travel history to India [14]. However, the origin of this Canadian strain remains unclear. None of the students in this study travelled to China or any other country and had no known exposure to any mumps patients. Therefore, the source of infection remains elusive. The cases in this study were not linked to any mumps outbreaks in Kota Kinabalu. Mumps is a notifiable disease in Malaysia and the Kota Kinabalu Area Health Office did not receive any mumps notifications before, during or after this outbreak. Since the infected students sought medical attention right away and their movement was restricted, the outbreak did not spread further.

This is the first study to report the occurrence of genotype F in Malaysia. Previously, only genotypes G and J were found circulating in Malaysia [11]. Cheng and Liu isolated a genotype G strain in Taiwan from a patient who travelled to Malaysia in 2006, implying that G MuVs circulated in Malaysia in 2006 [13]. Other Southeast Asian countries including Indonesia, Thailand, and Singapore also reported the occurrence of genotypes G and J but not genotype F [11, 13]. The genotype F strain found in this study may have been brought over to Sabah by travelers from regions with circulating genotype F MuV. Tourism is one of the important economic sectors in Sabah. Between 2013 and 2020, more than 50% of the international tourists arriving in Sabah were from East Asia, mainly from China [15]. Therefore, one possibility is that the cases involved in this outbreak somehow came into contact with tourists who may have been infected. Another possibility is that there is more widespread circulation of Genotype F MuV in Malaysia but due to lack of further investigation, its presence is not known.

Malaysia incorporated the trivalent measles-mumps-rubella (MMR) vaccine into the Malaysian National Expanded Programme of Immunisation in 2002 and began vaccination in 2004, wherein all children are given their first dose of the vaccine at 1 year of age and a second booster dose by 7 years of age [16, 17]. The live attenuated mumps vaccine strains used globally include Jeryl Lynn (genotype A), S79 (genotype A), RIT 4385 (genotype A), Leningrad-3 (genotype N), L-Zagreb (genotype N), Rubini (genotype A) and Urabe Am9 (genotype B) [18, 19]. Jeryl Lynn and Urabe Am9 were the most widely used strains in the world, including Malaysia [17, 20]. However, the production of the Urabe Am9 mumps vaccines was discontinued in 1993 due to an increased risk of meningitis in vaccinated children [19].

All the currently licensed MMR vaccines use strains that circulated predominantly in the pre-vaccination era, namely genotypes A and B [19, 20]. The efficacy of these vaccines could be reduced with the emergence of other genotypes besides A and B. Reports have suggested that neutralizing antibodies are specific to the vaccine strain used and humoral immunity is not adequately efficient in preventing infection by different MuV genotypes [21]. Despite the reduced effectiveness of the current vaccines due to antigenic differences between vaccine strains and contemporary circulating MuV genotypes, studies have shown that immunization with unmatched vaccine strains still effectively neutralized genetically different MuV strains [22, 23]. This study reports the detection of the first instance of genotype F MuV strain in Malaysia, where only genotypes G and J were circulating thus far, making this a great concern. These findings underscore the importance of studying and monitoring currently circulating MuV strains to improve the selection of vaccine strains used for maximum effectiveness. However, none of the students in this study were vaccinated against mumps and therefore no conclusions can be made about the reduced effectiveness of the current vaccine used in Malaysia.

The study has some limitations. There was a delay between the onset of outbreak and notification, resulting in a limited number of mumps cases for sample collection which reduced the number of samples for analysis. Therefore, the findings of this study cannot be generalized due to the small sample size. With the quick response from the Kota Kinabalu Area Health Office and the Faculty of Medicine and Health Sciences, Universiti Malaysia Sabah, cases were isolated and the outbreak did not spread further.

## Conclusions

The resurgence of this once-dormant disease, possibly due to waning immunity and unmatched vaccine strains [21], requires that relevant authorities reconsider strategies for the management of mumps. Results from this study revealing the first appearance of genotype F MuV in Sabah and Malaysia may assist local authorities in planning effective control and prevention strategies for mumps infection. Due to the limited number of samples in this study, further research with more samples from different areas in Malaysia is necessary to acquire deeper knowledge of the molecular features and etiology of mumps in the country.

## Data Availability

Data and materials are available from the corresponding author upon reasonable request.

## Abbreviations

cDNA: Complementary deoxyribonucleic acid
F: Fusion
HN: Haemagglutinin-neuraminidase
L: Large
M: Matrix
MMR: Measles-mumps-rubella
MuV: Mumps virus
NP: Nucleoprotein
P: Phosphoprotein
PCR: Polymerase chain reaction
RNA: Ribonucleic acid
RT-PCR: Reverse transcription polymerase chain reaction
SH: Small hydrophobic
V: Virion

## References

[1] Lam E, Rosen JB, Zucker JR (2020). Mumps: and update on outbreaks, vaccine efficacy, and genomic diversity. Clin Microbiol Rev.

[2] Kubota M, Hashiguchi T (2021). Unique tropism and entry mechanism of mumps virus. Viruses.

[3] Rubin SA, Plotkin SA, Plotkin SA, Orenstein WA, Offit PA (2013). Mumps vaccine. Vaccines.

[4] Connell AR, Connell J, Leahy TR, Hassan J (2020). Mumps outbreaks in vaccinated populations—is it time to re-assess the clinical efficacy of vaccines?. Front Immunol.

[5] World Health Organization. Mumps virus nomenclature update: 2012 = Nomenclature des virus ourliens: mise à jour 2012. Wkly Epidemiol Rec. 2012;87:217–224. https://apps.who.int/iris/handle/10665/241922.

[6] Mitui MT, Tabib SM, Matsumoto T, Khanam W, Ahmed S, Mori D (2012). Detection of human bocavirus in the cerebrospinal fluid of children with encephalitis. Clin Infect Dis.

[7] Jin L, Beard S, Brown DW (1999). Genetic heterogeneity of mumps virus in the United Kingdom: identification of two new genotypes. J Infect Dis.

[8] National Committee for Clinical Research. Malaysian guidelines on the use of human biological samples for research. Ministry of Health Malaysia. 2015. https://clinicalresearch.my/wp-content/uploads/2020/11/Guideline-on-Human-Tissue-in-Clinical-Research.pdf. Accessed 4 May 2020.

[9] Donahue M, Hendrickson B, Julian D, Hill N, Rother J, Koirala S (2020). Multistate mumps outbreak originating from asymptomatic transmission at a Nebraska wedding—six states, August–October 2019. MMWR Morb Mortal Wkly Rep.

[10] Feng D, Zhang L, Lyu W, Li G, Xu J, Zhang Y (2019). Genetic characteristics of mumps virus isolated from one outbreak in Henan province in 2016. Chin J Microbiol Immunol.

[11] Jin L, Örvell C, Myers R, Rota PA, Nakayama T, Forcic D (2015). Genomic diversity of mumps virus and global distribution of the 12 genotypes. Rev Med Virol.

[12] Cui A, Rivailler P, Zhu Z, Deng X, Hu Y, Wang Y (2017). Evolutionary analysis of mumps viruses of genotype F collected in mainland China in 2001–2015. Sci Rep.

[13] Cheng WY, Liu MT (2018). Molecular characteristics of mumps viruses isolated in Taiwan from 2006 to 2016. Heliyon.

[14] Nucleotide [Internet]. Bethesda (MD): National Library of Medicine (US), National Center for Biotechnology Information; [1988]. Accession No. MN911884.1, mumps virus genotype F strain MuVs/British Columbia.CAN/34.16/2 small hydrophobic protein (SH) gene, complete cds. 2020. https://www.ncbi.nlm.nih.gov/nuccore/mn911884.1. Accessed 10 Feb 2022.

[15] Goh HC (2021). Strategies for post-COVID-19 prospects of Sabah’s tourist market—reactions to shocks caused by pandemic or reflection for sustainable tourism?. Res Glob.

[16] Tan MS, Teoh EJ, Hor CP, Yeoh AAC (2016). Measles-Mumps-Rubella vaccine for children with egg allergy: Is admission for inpatient vaccination necessary?. Med J Malaysia.

[17] Ministry of Health Malaysia. Clinical practice guidelines: Childhood immunization (MOH/P/PAK/81.04(GU). 2004. https://www.moh.gov.my/moh/attachments/3934.pdf. Accessed 5 May 2020.

[18] Hao X, Wang Y, Zhu M, Zhou D, Liu R, Wang B (2021). Development of improved mumps vaccine candidates by mutating viral mRNA cap methyltransferase sites in the large polymerase protein. Virol Sin.

[19] Almansour I (2020). Mumps vaccines: current challenges and future prospects. Front Microbiol.

[20] Su SB, Chang HL, Chen KT (2020). Current status of mumps virus infection: epidemiology, pathogenesis, and vaccine. Int J Environ Res Public Health.

[21] Barrabeig I, Antón A, Torner N, Pumarola T, Costa J, Domínguez A (2019). Mumps: MMR vaccination and genetic diversity of mumps virus, 2007–2011 in Catalonia, Spain. BMC Infect Dis.

[22] Rubin SA, Qi L, Audet SA, Sullivan B, Carbone KM, Bellini WJ (2008). Antibody induced by immunization with the Jeryl Lynn mumps vaccine strain effectively neutralizes a heterologous wild-type mumps virus associated with a large outbreak. J Infect Dis.

[23] Rubin SA, Link MA, Sauder CJ, Zhang C, Ngo L, Rima BK (2012). Recent mumps outbreaks in vaccinated populations: no evidence of immune escape. J Virol.

